# Society Meetings

**Published:** 1914-06

**Authors:** 


### SOCIETY MEETINGS

Brief reports and announcements of meetings of societies of Western New York, and those of general scope, are requested from Secretaries. Copy should be on hand the fifteenth of the month. Full transactions will be published at cost of composition.

---

**The National Dental Association** will meet in Rochester, N. Y., on July 7, 8, 9, 10, and papers will be read by the following members of the medical fraternity: Dr. Grover W. Wende of Buffalo, "Syphilis in Dentistry"; Dr. Victor C. Vaughn, of Ann Arbor, "The Functions of Dentistry and Medicine in Race Betterment," and Dr. Joseph C. Bloodgood, of Baltimore, "Early Recognition of Precancerous Lesions of the Mouth and Tongue."

---

**The American Proctologic Society** will meet at Atlantic City June 22 and 23, headquarters at the Chalfonte.

---

**The American Gastro-Enterologic Association** will meet on the same dates.

---

**The American Society for Physicians' Study Travels** will make its first annual tour, June 26 to July 16, starting from Atlantic City. Various entertainments, sight seeing and clinics will be held in Philadelphia on Saturday. On Sunday, June 28, there will be an excursion to League Island Navy Yard. On Monday, the Society will be entertained at White Haven Sanitarium. Tuesday, June 30, and Wednesday, July 1, will be spent at Buffalo, visiting the State Institution for Malignant Diseases and various local points of interest. From Buffalo, the Society goes to Niagara Falls, Toronto, Montreal, Quebec, Portland, Boston, Saranac Lake, Saratoga Springs,

Albany and thence by boat to New York, the last two days being devoted to clinics and research work.

At the time of writing, details for the Buffalo entertainment have not been completed. A number of automobiles will be needed for a trip about the city and physicians are requested to telephone offers to Dr. Charles G. Stockton or to the editor.

---

**Rochester Academy of Medicine,** Section 1 on General Medicine, etc., met May 14. Dr. G. Kirby Collier, Assistant Superintendent of Craig Colony gave a paper on the treatment of Epilepsy, the discussion being led by the Superintendent, Dr. W. T. Shanahan.

---

A special meeting of the Academy was held May 6, at the Genesee Valley Club, Dr. Joel E. Goldthwaite, of Boston, presenting a paper on "The Anatomic and Mechanistic Conception of Disease."

---

**The Alumni Association of the Medical Department of the University of Buffalo** held it annual meeting, June 2, 3, 4, 5. As the meeting occurred during the time that this issue was on the press, it was too late for an advance notice and too early for a report. The proceedings will, therefore, be published in the July issue.

---

**The Western New York Homoeopathic Society** held its 30th annual meeting in Buffalo, May 1, at the Homoeopathic Hospital. The banquet was held at the Hotel Statler in connection with the Western New York alumni of the New York Homoeopathic Medical College.

---

**The Buffalo Academy of Medicine** has held the following meetings since the last published report: April 28, Section of Surgery, Principles Underlying the Transplantation of Fascia, Tendon and Bone, Dr. Dean Lewis of Rush Medical College, Chicago. May 5, Section of Medicine, Something of Eugenics, Dr. H. W. Cowper; Diagnosis and Treatment of Diphtheria, Dr. W. S. Goodale, the latter discussed by Drs. H. C. Buswell, N. G. Russell and W. G. Bissell. May 12, Section of Obstetrics and Gynaecology, Treatment of Puerperal Infection, Dr. R. R. Huggins, of Pittsburgh. This was illustrated by lantern slides. The discussion opened by Dr. W. Mortimer Brown of Rochester and Dr. Irving W. Potter of Buffalo. May 19, Section of Pathology, Haemoptysis in Childhood with presentations of specimens, Dr. Carl G. Leo-Wolf, The Wassermann Test in the Municipal Laboratory and the interpretation to be placed on results, Dr. Wm. G. Bissell. Discussion by Drs. Grover W. Wende, H. C. Buswell, and A. A. Thibaudeau.

---

**The A. M. A.** will meet at Atlantic City, June 22, 23, 24, 25, 26. For the program and other announcements, we refer to the Journal of the A. M. A. of May 16. We want to say a few words about railroad rates, however. With minor details differing for the geographic associations of railroads, the rate granted is two cents per mile, each way, with a time limit of June 20, 21, 22, going, and June 29, returning. This is the same for mileage books, the latter being good for a year but sold only by thousands or half thousands of miles. It is about the same as for a regular six-months excursion ticket. It is about seven dollars more for Buffalo physicians than the rate granted in 1900 or than the rate granted to pretty nearly every other body of men or for special excursions gotten up by the railroads themselves at various times. Taken in connection with the refusal of a special rate to the recent state convention in New York City, this action on the part of the railroads should arouse concerted and vigorous action on the part of the medical profession.

---

**The Interurban Orthopedic Club** met at Rochester, N. Y., May 1st and 2d, 1914. The program was as follows:

Specialism and the Common Good — Dr. L. W. Jones
Remarks Concerning the Rochester Infants Summer Hospital — Dr. Norris G. Orchard
Feeding in Chronic Arthritis — Dr. Chas. R. Witherspoon
Metabolism and Disease — Dr. C. Clyde Sutter
The Relations Between the Ductless Glands and Cancer — Dr. Seelye W. Little
Claw Foot, Remarks on Birth Paralysis, Congenital Elevation of the Scapula, Scoliosis — Dr. Howard L. Prince

1:30 P. M. Luncheon at the Genesee Valley Club

3:00 P. M. Taylor Instrument Company

Demonstration of the Electro-Cardiograph and Einthoven String Galvanometer — Dr. Edward W. Jackson

4:30 P. M. Country Hospital for Crippled Children
Dr. Ralph R. Fitch

7:00 P. M. Dinner

Saturday, May 2, 10 o'clock, Rochester General Hospital

Demonstration of the New Coolidge X-Ray Tube — Prof. J. S. Shearer of Cornell University
A Case of Refracture of the Femur — Dr. C. Wentworth Hoyt
Fractures About the Elbow Joint — Dr. Myron B. Palmer
A Comparison Between the Wassermann and Noguchi Reactions — Dr. Michael L. Casey

A syringe for Intravenous Injection — Dr. Joseph Roby
Remarks on Transfusion — Dr. Charles W. Hennington
A Portable Gas-Pipe Traction Spica Frame
A Modified Bradford Frame
A Modified Goldthwait Frame
The Hawley Operating Table
A Means of Obtaining Traction in Cases of Marked Thigh Flexion, Associated with Extreme Deformity of Pott's Disease
A Ball Bearing Roller Joint
Arthrodesis of the Acromio-Clavicular Articulation
Giant Cell Sarcoma
Some Problems in Adult Backs — Dr. Ralph R. Fitch

1:30 P. M. Luncheon at the Rochester Country Club

---

**The Medical Society of the County of Monroe** held its annual meeting, May 19. Clinics were held in the morning as follows:

1. General Hospital. Medicine. 9 A. M.
   Dr. Joseph Roby in the medical wards on diagnostic and therapeutic procedures including Tuberculin Reactions, Schlick Diphtheria Reaction, Intravenous and Intraspinous Injections, Artificial Pneumothorax.
2. General Hospital Surgery. 9 A. M.
   Dr. E. W. Mulligan in the operating rooms. Attendance limited in the order of reply post cards received. Dr. C. N. Jameson to demonstrate NO-O Anaesthesia.
3. General Hospital. Obstetrics. 10 A. M.
   Dr. W. M. Brown in the Hart Maternity Building, on Obstetrical Diagnosis, Abdominal Palpation and Pelvimetry.
4. General Hospital. Orthopedics. 10 A. M.
   Drs. Fitch and Prince on the fourth floor of West Hall on diagnosis of various orthopaedic conditions. Also, application of Abbott Jacket for correction of Lateral Curvature of Spine.
5. General Hospital. Roentgenology. 10 A. M.
   Dr. Palmer in the X-Ray Room on the second floor of the Administration Building, on the Gastro-Intestinal Tract.
6. General Hospital. Pathology. 10 A. M.
   By staff in the Laboratory on the second floor of the Administration Building, including especially, Drs. Boswell and Costello on Wasserman Reactions and Dr. Sutter on the Collodial Gold Reaction of Spinal Fluid.
7. General Hospital. Dermatology. 9 to 11 A. M.
   Dr. Roseboom in the Out-Patient Department.

8. General Hospital. Eye, Ear, Nose and Throat. 9 to 11.
Drs. L. W. Jones and A. G. Morris, and Drs. Ingersoll and McDowell, In Out-Patient Department.

9. St. Mary's Hospital. Surgery. 9 A. M.
Dr. O. E. Jones in the Operating Room.

10. St. Mary's Hospital. Orthopaedics and Roentgenology. 11 A. M.
Drs. L. A. Whitney and L. R. Cornman.

11. Park Avenue Hospital. Surgical Clinic. 9:30 A. M.
Dr. C. R. Barber

12. Municipal Hospital. Infectious Diseases. 11 A. M.
Dr. G. W. Goler.

13. State Hospital. Psychiatry. 10 A. M. By the Staff.

14. County Hospital. Chronic Diseases. 10 A. M.
Dr. L. J. Somers.

15. Iola Sanitarium. Tuberculosis. 10 A. M.
Drs. Leary and Brayton.

16. Rochester Public Health Association. Diagnosis of Tuberculosis. 9 A. M.
Dr. Whipple.

17. Rochester Public Health Association. Dermatology. 10 A. M.
Dr. Landauer.

18. Rochester Public Health Association. Neurology. 11 A. M.
Dr. VanderBeek.

19. Open-Air School. 10 A. M.
Dr. Aikman. Inspection of the New Buildings on Culver Road near the Widewaters. The Medical Phases of the work.

20. On the Electro-Cardiograph. 10 A. M.
Dr. Jackson. A practical demonstration of the Electro Cardiographic Method in Heart Diagnosis at the Taylor Brothers Co., on Ames Street, near West Avenue.

After luncheon at the Powers Hotel a business session was held and the following program was presented:

Practical Orthopaedic Points for the Guidance of the General Practitioner.
Dr. James Warren Sever, Instructor in Orthopaedic Surgery, Harvard Medical School.

Disturbances of the Internal Secretions.
Dr. Thomas McCrae, Professor of Medicine, Jefferson Medical College.

Discussion of Medical Economics and Ethics.

